# Preparation and Desalination Performance of PA/UiO-66/PES Composite Membranes

**DOI:** 10.3390/membranes11080628

**Published:** 2021-08-16

**Authors:** Dai Xuan Trinh, Ngo Nghia Pham, Patchanee Chammingkwan, Toshiaki Taniike

**Affiliations:** 1 Faculty of Chemistry, VNU University of Science, Vietnam National University, 19 Le Thanh Tong, Hoan Kiem, Hanoi 10000, Vietnam; phamngonghia@hus.edu.vn or; 2 Institute of Environmental Engineering and Management, University of Witten/Herdecke, Alfred-Herrhausen-Straße 44, 58455 Witten, Germany; 3 Graduate School of Advanced Science and Technology, Japan Advanced Institute of Science and Technology, 1-1 Asahidai, Nomi 923-1292, Ishikawa, Japan; chamming@jaist.ac.jp (P.C.); taniike@jaist.ac.jp (T.T.)

**Keywords:** polymeric membrane, metal-organic framework membrane, UiO-66, nanochannels

## Abstract

UiO-66 nanoparticles are considered highly potential fillers for the application in desalination membranes. In this study, UiO-66 nanoparticles were anchored to PES membrane substrates, which were subsequently subjected to the interfacial polymerization reaction to coat a layer of polyamide (PA) on their surface. For comparison, a blank membrane incorporating no UiO-66 and a reference membrane incorporating ZrO_2_ (instead of UiO-66) were prepared. All prepared membranes were tested for their desalination performance. The membranes containing UiO-66 were found to outperform the blank and the reference counterparts. The reason for this outperformance is possibly attributed to the hydrophilicity of UiO-66 nanoparticles and the presence of nanochannels in their structure.

## 1. Introduction

Materials possessing uniform nanoscale channels are considered next-generation candidates for the application in filtration membranes, for example, carbon-based materials (graphene, graphene oxide, and carbon nanotube), protein-based counterparts (Aquaporin), and metal-organic frameworks (UiO-66 nanoparticles) [1]. In several cases, the pore structure of these materials can be conveniently tuned during the preparation procedure to obtain the desired properties, which additionally increases their attractiveness [2]. Several materials demonstrated superior permeability and selectivity during the filtration process. For instance, membranes consisting of graphene in a single-layer form with pore sizes between 0.5 and 1 nm, which were obtained in the study of Surwade et al., demonstrated remarkable water flux with a salt rejection efficiency of nearly 100% [3]. In another study carried out by Holt et al., membranes that incorporate carbon nanotubes of sizes less than 2 nm exhibited a water flow rate 1000 times higher than values calculated from the theoretical model [4]. In the study of Kumar et al., the incorporation of Aquaporin Z into the matrix of amphiphilic triblock-polymer membranes improved its water permeability by up to 800 times, which reached a value that is at least an order of magnitude higher than that of current conventional reverse osmosis membranes [5]. 

Concerning the metal-organic framework (MOF), different candidates have been successfully employed until recently [6,7,8]. In the study of Sotto et al., membranes prepared from MOF-74 and polyethylene sulfone (PES) displayed higher permeability and antifouling properties than the neat PES membrane [9]. In another study, Li et al. prepared a layer of ZIF-8 on the PES substrate giving membranes that demonstrated remarkable selectivity [10]. In a study performed by Ma et al., UiO-66, one of the most stable MOFs, was anchored into a graphene oxide (GO) structure before being dispersed into the matrix of the polyethersulfone membrane [11]. The incorporation of the UiO-66@GO into the structure of the polyethersulfone membrane increased its water flux by more than 3.5 times. More recently, Liu et al. coated UiO-66 on the outermost surface of alumina hollow fiber by the secondary growth method, giving a 2-μm-thick layer that could reject multivalent cations (98% for Mg^2+^ and 99.3% for Al^3+^) from the water via size exclusion mechanism [12]. 

The superior performance of the MOF materials in filtration membranes had motivated us to carry out different studies in the past [13,14]. In a study performed in 2017, we anchored UiO-66 nanoparticles into the matrix of regenerated cellulose membranes during the preparation of nanofiltration membranes. The resulting membranes, which allowed the transport of water molecules preferentially through the intraparticle channels of UiO-66 nanoparticles, demonstrated a perfect selectivity of methylene blue from aqueous solution while maintaining excellent permeability and flexibility. However, after these studies, we recognized a potential drawback during the incorporation of MOF materials on the surface of the substrate membrane. This type of deposition can result in the formation of non-selective interparticle void among UiO-66 nanoparticles that might eventually cause leakages of small solutes. Therefore, we have considered a strategy to overcome this drawback, for which we report the preliminary results in this paper, by preparing polyamide-polyethersulfone membranes deposited with UiO-66 nanoparticles via a three-step process. Initially, UiO-66 nanoparticles were coated on the surface of the PES substrate membrane via suction filtration. After that, the UiO-66 embedded substrate membrane was subjected to an interfacial polymerization reaction, during which the immersion times of the membranes in aqueous and organic solutions were varied to obtain barrier layers of different thicknesses [15,16]. To understand the effect of UiO-66 nanoparticles on the properties of the material, a blank membrane incorporating no UiO-66 nanoparticles and a control membrane incorporating ZrO_2_ (instead of UiO-66) were prepared following analogous procedures. Finally, all prepared membranes were tested for their desalination performance, and their performance results were compared. To the best of our knowledge, compared to the previously reported works given in Table 1 [17,18,19,20,21,22,23,24,25], the three-step approach of this study that employs UiO-66 nanoparticles has not been published yet.

## 2. Materials and Methods

### 2.1. Materials

Zirconium tetrachloride (ZrCl_4_) (purity > 99.9%) and terephthalic acid (purity > 99%) were purchased from Sigma-Alrich. N,N-dimethylformamide (DMF) was obtained from Wako Chemical Industries Ltd. (Richmond, VA, USA). Polyethersulfone membrane (diameter 47 mm, pore size 0.22 µm) was received from Millipore. 1,3-Diphenylene diamine (MPD) (purity > 98%), trimesoyl chloride (TMC) (purity > 98%), trimethylamine (TED) (purity > 99%), and (+)-10-camphosulphonic acid (CSA) (purity > 98%) were bought from Tokyo Chemical Industry Co. Ltd. (Tokyo, Japan). Hexane (purity > 96%) was delivered from Kanto Chemical Co. Inc. (Tokyo, Japan) These chemicals were used without further purification.

### 2.2. Preparation of UiO-66 Nanoparticles

The preparation of UiO-66 nanoparticles was carried out under a nitrogen atmosphere following our previously documented procedure [13]. Briefly, ZrCl_4_ (0.30 g) was dissolved in 90 mL of DMF. At the same time, another solution was prepared, which contained 0.22 g of terephthalic acid dissolved in 90 mL of DMF. Thereafter, both solutions were mixed and stirred. To the mixture, 0.18 mL of water were added. The water acted as a modulator that raises the nucleation rate and controls the distribution size of UiO-66 nanoparticles. Subsequently, the mixture was stirred at 100 °C for 20 h, giving a homogeneous and stable dispersion of UiO-66 nanoparticles in DMF. UiO-66 nanoparticles were immersed in methanol for three days. During this time, the solvent was removed, and an identical amount of pure methanol was compensated every day. Prior to the preparation of UiO-66/PES membranes, the UiO-66-containing methanol solution was sonicated for 2 h followed by centrifugation at 2000 rpm for 10 min. Finally, the supernatant was used for the fabrication step of the UiO-66/PES membrane.

### 2.3. Membrane Preparation

UiO-66/PES membranes were prepared by a suction filtration set-up following the previously reported procedure [13]. Briefly, a PES support membrane was placed on a filter holder. Then, 0.5 mg of UiO-66 nanoparticles dispersed in 0.25 mL of methanol were dropped on the top of the PES membrane at a differential pressure of 20 mbar before the membrane was washed with 10 mL of distilled water. After that, the membrane was taped on a glass plate with the UiO-66 side exposed and soaked in an aqueous solution mixture of MPD, CSA, and TEA. After a predetermined time, the glass plate was taken out. The excess liquid on the membrane was removed using nitrogen flow. After that, the membrane was soaked in a solution of 0.1% of TMC in hexane for a predetermined time. Finally, it was cured at 70 °C for 30 min to obtain membranes M2-8 (Entries 2–8, Table 2). The reference membranes M0, M1, and M9 (entries 0, 1, and 9, Table 2) were prepared by adapting the procedure for the fabrication of PA/UiO-66/PES membranes M2-8. These membranes were stored in water until being used or characterized. 

### 2.4. Characterizations

The functional groups of the membrane surfaces were analyzed by attenuated total reflectance infrared spectroscopy (ATR-IR, Perkin Elmer Spectrum 100 FT-IR (Waltham, MA, USA) in the range 450–1800 cm^−1^ using a diamond crystal. Transmission electron microscopy (TEM, Hitachi H7100, Tokyo, Japan) at an acceleration voltage of 100 kV was used to determine the size and morphology of UiO-66 nanoparticles. The UiO-66 dispersion was diluted 100 times in methanol and casted onto a TEM grid. The crystalline structure of the dried UiO-66 nanoparticles was analyzed by X-ray diffraction (XRD, Rigaku SmartLab, Austin, TX, USA) using Cu Kα radiation (λ = 1.54 Å) at 40 kV and 30 mA in the interval 5–35°.

The morphology of the membranes was observed by scanning electron microscopy (SEM, Hitachi S-4100, Tokyo, Japan) at an accelerated voltage of 20 kV. The hydrophilicity of the membrane was evaluated by measurement of the contact angles on a contact angle meter (Dropmaster DM-501, Kyowa Interface Japan, Saitama, Japan).

### 2.5. Desalination Tests

**Figure 1:**
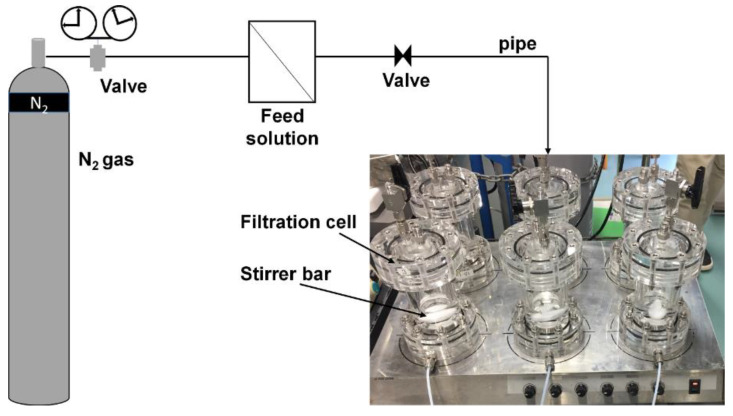
Setup of the desalination test in this study.

The desalination tests were conducted using six identical handmade dead-end stirred cells (Figure 1), which were connected to a tank of the feed solution (1000 ppm of NaCl in deionized water). A membrane was placed in the filtration holder and preconditioned by filtering distilled water for 1 h at a differential pressure of 3.0 bar. After that, the feed solution was stirred and filtered at a differential pressure of 2.0 bar. The pressure was generated by a nitrogen cylinder equipped with a pressure regulator. The concentration of NaCl in the feed and permeate solutions was determined by a conductivity meter.

The filtration system was comprised of 6 identical filtration cells (Figure 1). A filter holder was designed for a maximal pressure of 10 bars, feed volume per cell of 100 mL, and an effective membrane area of 7.1 cm^2^. Each filtration cell was equipped with a stirred bar to avoid concentration polarization during filtration. 

The permeability (J) and the selectivity (R) were determined via the following equation:

(1) $$J=\frac{V}{S\cdot \Delta P\cdot t}$$

(2) $$R=\frac{C_{f}-C_{p}}{C_{f}}\cdot 100$$

where V, S, ΔP, and t represent the permeate volume, effective area of the membrane, differential pressure, and filtration time, respectively, whereas C_f_ and C_p_ represent the concentration of NaCl in the feed and permeate solution, respectively.

## 3. Results and Discussions

### 3.1. Characterization of UiO-66 Nanoparticles

**Figure 2:**
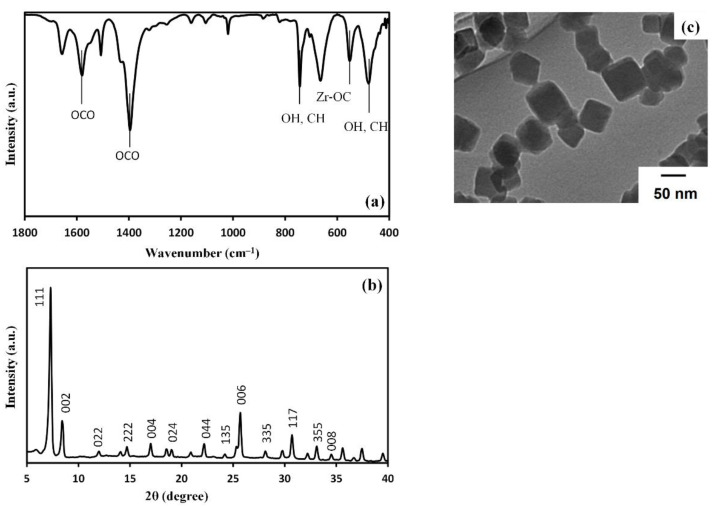
Characterization of UiO-66 nanoparticles: (**a**) FT-IR spectrum, (**b**) XRD diffractogram, and (**c**) TEM image of UiO-66 nanoparticles.

The ATR-IR spectrum of UiO-66 nanoparticles is depicted in Figure 2a. The peaks at 1574 and 1395 cm^−1^ were ascribed to the out-of-phase stretching modes of the carboxylate group. On the other hand, the peaks at 475, 548, and 744 cm^−1^ are attributed to the bending of OH and CH mixed with Zr-O modes as well as Zr-(OC) asymmetric stretching vibration, respectively. The absence of bands assigned to the possible remaining solvents indicates a thorough washing process. Overall, these peaks are in accordance with previously reported results [26].

The X-ray diffractogram of UiO-66 is illustrated in Figure 2b. It indicates the presence of a face-centered cubic (fcc) structure. The peaks at 7.26, 8.39, 14.02, and 14.64° are assigned to the (111), (002), (022), and (222) planes, respectively [27,28]. 

The TEM image of the UiO-66 nanoparticles is given in Figure 2c. As can be seen, UiO-66 nanoparticles show a cubic-shaped morphology with an average size of 50 nm. The shape and size of UiO-66 nanoparticles were reported to depend on the amount of water in the reaction solution. In these reports, water was supposed to modulate the nucleation rate and thus the growth of Zr_6_O_4_(OH)_4_ clusters.

### 3.2. Characterization of the Prepared Membranes

**Figure 3:**
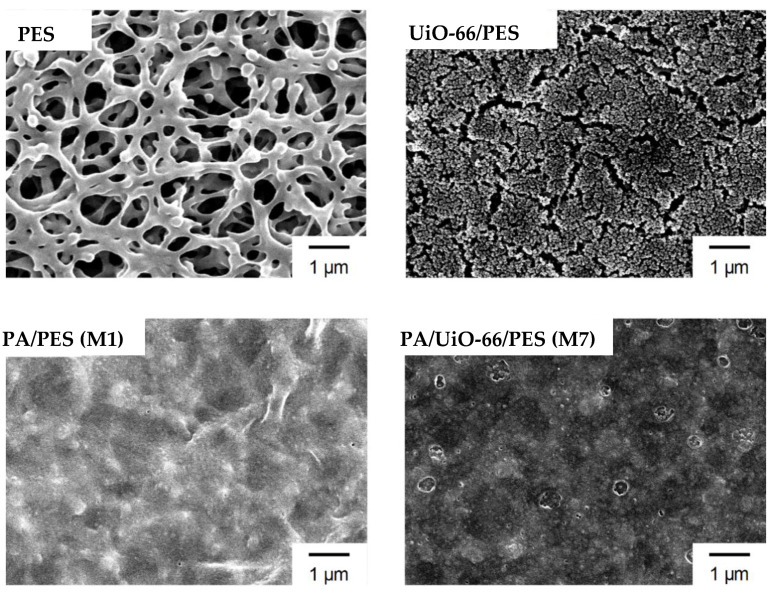
SEM images of the membranes in this study.

The prepared membranes were characterized by SEM analysis (Figure 3). The PES support membranes show pores of approximately 0.2 µm in size, which is suitable for the microfiltration application. The deposition of UiO-66 nanoparticles in the matrix of the PES membrane was clearly observed. The formation of cracks on the surface was attributed to the thermal stress during the SEM measurement process. The SEM results display a dense morphology of the surface of the blank membrane M1 and the UiO-66-coated membrane M7. 

The wettability of the prepared membranes was evaluated by measurement of the water contact angle (WCA). WCA of the neat PA/PES membrane M1 was 64°. In comparison, WCA of the UiO-66-coated membranes M7-8 was 55.5 and 54.4°, respectively. It indicates that the incorporation of the hydrophilic UiO-66 nanoparticles improved the hydrophilicity of the PA/PES membrane. Additionally, the higher the concentration of UiO-66 nanoparticles inside the membrane matrix, the higher the hydrophilicity of the PA/PES membrane. Generally, the higher hydrophilicity of the membrane is expected to demonstrate higher permeability [29]. 

### 3.3. Desalination Performance of the Prepared Membranes

**Figure 4:**
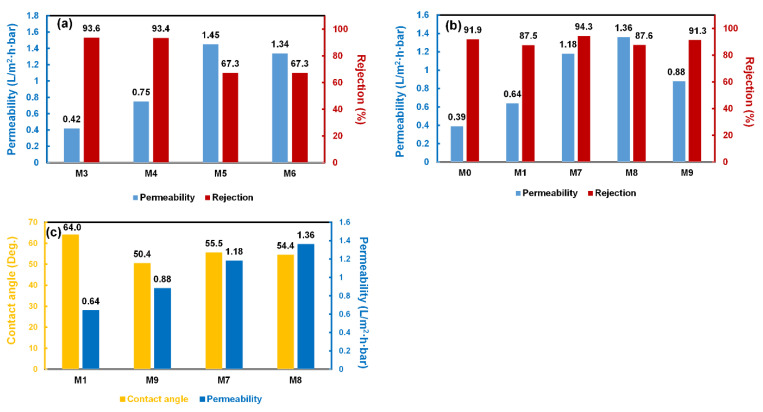
Filtration performance of the prepared and reference membranes: (**a**,**b**) the permeability and rejection, and (**c**) the contact angles and permeability of the membranes.

First, the blank membrane M1 was tested for desalination. M1 has a WCA value of 64°. It displays a permeability of 0.64 L∙m^2^∙h∙bar and a selectivity of 87.5% (Figure 4b). In comparison, the UiO-66 nanoparticle-deposited membrane M2 (Entry 2, Table 2) has higher wettability with a WCA value of 55.5°. Materials with a WCA value of less than 90° are considered hydrophilic. Generally, the lower the WCA value is, the higher the wettability the membranes display. The wettability is proportional to the surface roughness and surface hydrophilicity [30]. The incorporation of the hydrophilic UiO-66 nanoparticles increases the surface roughness (Figure 3) and surface hydrophilicity of the membrane. During the desalination test, membrane M2 demonstrated a permeability of 0.31 L∙m^2^∙h∙bar with a selectivity of 90.2%. Compared to the blank PA/PES membrane M1, the UiO-66-deposited membrane M2 showed a lower permeability (50% lower) but a higher selectivity. Hence, the presence of UiO-66 nanoparticles improved the selectivity. This improvement was referred in previous studies to the facilitation of mass transport through the nano-sized channels of the UiO-66 nanoparticles [31,32,33]. 

In the next step, we attempted to find more suitable conditions for preparing membranes with higher permeability. We reduced the thickness of the barrier layer by shortening the immersion time of the membrane with the organic phase from 60 to 15 s during the fabrication of membrane M3 (Entry 3, Table 2). Fortunately, the permeability increased to 0.42 L∙m^2^∙h∙bar. Noticeably, the selectivity also increased to 93.6% (Figure 4a). 

Subsequently, we attempted to reduce the number of the amine monomers on the surface of the membrane by reducing the contact time with the aqueous solution from 300 to 120 s (Entry 4, Table 2). Fortunately, membrane M4 exhibited a significantly higher permeability (0.75 L∙m^2^∙h∙bar) compared to membrane M3 (0.42 L∙m^2^∙h∙bar). The permeability of membrane M4 is even higher than that of the blank membrane (0.64 L∙m^2^∙h∙bar) (Figure 4a). It is a strong indication for the hypothesis that the UiO-66 nanoparticles not only improve the selectivity but also facilitate the transport of the molecules due to their nano-sized channels [34]. 

Consequently, we reduced the density of the polyamide network by reducing both the MPD concentration and contact time with the organic phase (Entry 5, Table 2). Although membrane M5 showed a remarkably higher permeability (1.45 L∙m^2^∙h∙bar), it lost more than 25% of the selectivity due to the insufficient presence of UiO-66 nanoparticles and the integrity of the selective layer. Therefore, we increased the integrity of the barrier layer by raising the contact time with the organic phase to 10 s (Entry 6, Table 2), giving membrane M6, which did not show a better performance, unfortunately. Finally, we attempted to further increase the thickness of the barrier layer by increasing the contact time with the organic phase to 15 s. Fortunately, we obtained membrane M7 with a lower permeability (1.18 L∙m^2^∙h∙bar) but remarkably higher selectivity (94.3%) (Figure 4b). Further effort to increase the amount UiO-66 nanoparticles resulted in membrane M8 having a higher permeability (1.36 L∙m^2^∙h∙bar) but lower selectivity (90.5%) (Entry 8, Table 2). The experimental results show that M7 possessed the best barrier layer among the experiment entries, due to the loading of a suitable amount of UiO-66 nanoparticles and the integrity of the PA layer. Increasing the loading of UiO-66 nanoparticles on the PA layer caused the formation of defects in the structure of the barrier layer, possibly due to the incompatibility at the boundary between the polyamide and UiO-66 nanoparticles. Consequently, the permeability of M8 increased while showing poor selectivity. In comparison to the filtration performances between the blank and PA/UiO-66/PES membranes, Figure 4b shows that in the same preparation conditions (M1 and M7), the permeability of M7 was almost two times higher than that of M1 while it possessed better salt rejection. At a similar rejection (M0 and M8), UiO-66 nanoparticles increased three times the permeability.

Several studies agree that the presence of UiO-66 nanoparticles potentially facilitates the transport process owing to their nanopore channels [13,35]. To assess this statement, we applied identical preparation conditions from UiO-66 nanoparticles for ZrO_2_ nanoparticles (M9, Entry 9, Table 2). Compared to UiO-66, ZrO_2_ nanoparticles possess no nanopore channels. Noticeably, compared to UiO-66-deposited membranes M2-8, membrane M9 demonstrated higher hydrophilicity (WCA value of 50.4°) but a lower permeability (0.88 L∙m^2^∙h∙bar) (Figure 4c). This is possibly attributed to the presence of the pore structure of UiO-66 nanoparticles, which facilitate water transport through their channels [36]. Notably, the size of these channels is approximately 6 Å [37]. This size allows a sufficient efficiency of the separation via ionic sieving [38]. Furthermore, the small size of the pore system potentially prevents the blockage of the pores by the polymers during the polymerization reaction, thus maintaining a good permeability [39].

## 4. Conclusions

To summarize, different PA/UiO-66/PES membranes were prepared by a three-step process. The membranes were characterized by the SEM technique, displaying a homogenous distribution of the particles on the membrane surface. The membranes were also tested for their desalination performance, giving promising filtration results. The membranes incorporating UiO-66 nanoparticles outperformed the blank PA/PES membrane and the reference PA/ZrO_2_/PES membrane. The improvement is possibly due to the hydrophilicity of UiO-66 nanoparticles and the stronger preference of water diffusion through the intra-particle channels. Additionally, the preference is possibly related to the compactness and thickness of the polyamide polymer, which depends on the amount of the monomer and the cross-linker adsorbed on the surface of the substrate membrane. Further investigations into this approach are currently being undertaken for other amine monomers and cross-linkers. 

## Figures and Tables

**Table 1:** Previously published works concerning nanoparticle embedded desalination membranes.

Nanoparticles	Intraparticle Channels	Feed Solution mg·L^−1^	Permeability Increase	NaCl Rejection (%) ^c^	Ref.
CeO_2_ NPs ^a^	without	2000	1.50	98.7–98.0	[17]
ZIF-8 ^a^	with	2000	2.07	98.1–99.5	[18]
CNTs ^a^	with	2000	1.18	97.5–95.4	[19]
Ag NPs ^b^	with	2000	2.68	97.4–99.1	[20]
SiO_2_^a^	with	2000	2.24	90.7–91.1	[21]
NaA zeolite ^a^	with	2000	1.63	91.4–98.0	[22]
GO ^a^	with	1000	1.76	94.0–94.8	[23]
UiO-66 ^b^	with	1000	3.02	91.9–94.3	this work

^a^ Two-step approach: Nanoparticles were dispersed in the aqueous phase or the organic phase for the interfacial polymerization. ^b^ Three-step approach: Nanoparticles were preloaded on the substrate membrane before the interfacial polymerization process. ^c^ Salt rejection of membranes: (left) membrane without nanoparticles, and (right) nanoparticles-loaded membrane.

**Table 2:** Membranes being prepared in this study.

Entry	Membrane	MPD Concentration (wt%)	UiO-66 Loading (mg)	Contact Time in Aqueous Phase (s)	Contact Time in Organic Phase (s)
0	PA/PES (M0)	3	0	120	15
1	PA/PES (M1)	1	0	120	15
2	PA/UiO-66/PES (M2)	3	0.5	300	60
3	PA/UiO-66/PES (M3)	3	0.5	300	15
4	PA/UiO-66/PES (M4)	3	0.5	120	15
5	PA/UiO-66/PES (M5)	1	0.5	120	5
6	PA/UiO-66/PES (M6)	1	0.5	120	10
7	PA/UiO-66/PES (M7)	1	0.5	120	15
8	PA/UiO-66/PES (M8)	1	1.0	120	15
9	PA/ZrO_2_/PES (M9)	1	0.5 (ZrO_2_)	120	15

## Data Availability

Not applicable.
